# Diurnal variation in brain injury after cardiac arrest and cardiopulmonary resuscitation

**DOI:** 10.3389/fneur.2025.1497046

**Published:** 2025-02-07

**Authors:** Fei Peng, Fei Wang, Bowen Gao, Ping Sun

**Affiliations:** ^1^Department of Anesthesiology, Sichuan Provincial People's Hospital, University of Electronic Science and Technology of China, Chengdu, China; ^2^Teaching Center for General Courses, Chengdu Medical College, Chengdu, China; ^3^Department of Orthopedic Anesthesiology, The Second Affiliated Hospital of Air Force Medical University, Xi'an, China

**Keywords:** circadian rhythm, diurnal, cardiac arrest, brain injury, neurological outcomes, ischemia–reperfusion

## Abstract

**Background:**

Although the circadian rhythm is known to influence several neurological diseases and response to treatments, its potential impact on brain injury following cardiac arrest and cardiopulmonary resuscitation (CA/CPR) remains unclear.

**Methods:**

We performed a retrospective observational study on out-of-hospital cardiac arrest (OHCA) cases that presented to the emergency department of our hospital between September 2022 and August 2024. Based on the CA/CPR onset time, all patients were divided into two cohorts: daytime and nighttime groups. The gray-to white-matter signal intensity ratio (GWR) was analyzed using brain computed tomography (CT) images. We used the Cerebral Performance Category (CPC) to estimate the neurological outcomes. C-reactive protein (CRP), white blood cell (WBC) count, and monocyte (MONO) count levels in the plasma were also analyzed.

**Results:**

Our study included 138 patients, of whom 68 were subjected to CA/CPR during daytime (8:00 to 20:00) and the remaining 70 were subjected to CA/CPR during nighttime (20:00 to 8:00). The imaging data showed that GWR values were significantly lower among patients subjected to CA/CPR during nighttime compared to those who were subjected to CA/CPR during daytime. Consistently, lower survival rates were observed among nighttime CA/CPR survivors. The CPC results indicated that a greater number of patients who underwent CA/CPR during daytime were rated as class 1–2 on day 3, day 5, and day 7 after achieving return of spontaneous circulation (ROSC). In contrast, a larger proportion of CA/CPR survivors in the nighttime group were rated as class 5 at the same time points. Elevated levels of C-reactive protein, white blood cell count, and monocyte count were observed in the plasma of survivors who underwent nighttime CA/CPR.

**Conclusion:**

We found that patients subjected to CA/CPR during nighttime (20:00–8:00) had worse neurological outcomes compared to those treated during daytime (8:00–20:00).

## Introduction

Cardiac arrest (CA) results in the sudden cessation of cardiac output and oxygen supply to the brain, which continues until reperfusion is achieved through cardiopulmonary resuscitation (CPR). Brain ischemia–reperfusion (I/R) injury occurs sequentially during cardiac arrest, resuscitation, and the acute post-resuscitation phase (1). Although early initiation of high-quality CPR increases the odds of improved neurological outcomes among CA patients, more than 60% of these patients still die from brain injury after cardiac arrest and cardiopulmonary resuscitation (CA/CPR) (2). Therefore, brain injury remains to be the main cause of mortality and morbidity among CA survivors (3).

A circadian rhythm is a 24-h endogenous cycle that regulates physiological functions, which helping organisms adapt to daily environmental changes (4). In recent decades, emerging evidence has suggested that the circadian rhythm could affect the pathogenesis of several neurological diseases and the response to treatments, such as epilepsy (5), depression (6), autism (7), and traumatic brain injury (8). Interestingly, previous studies have also unveiled the impact of the circadian rhythm on brain I/R injury. For example, Lu et al. revealed that the circadian rhythm influences the infarction volume and neurological outcomes in a mouse model of ischemic stroke (9). Clinically, the infarction volume is smaller among patients who experience ischemic stroke during the day compared to those who experience it during the night (10). In addition, the results of a systematic review suggested that the survival rate following CA is lowest during midnight (11). The aforementioned studies suggest that the circadian rhythm could affect neurological outcomes in patients affected by brain I/R injury.

However, whether the circadian rhythm truly influences brain injury after CA/CPR is still unknown. Gaining this knowledge could help adapt the design of CA/CPR trials and suggest new directions for research. In this study, we explored whether CA/CPR-induced brain injury shows diurnal variation by retrospectively analyzing patients with out-of-hospital cardiac arrest (OHCA). This updated understanding could influence treatment plans aimed at reducing the OHCA burden or improving survival rates after OHCA.

## Materials and methods

### Design and study population

This was a single-center, retrospective observational study conducted at Sichuan Provincial People’s Hospital, University of Electronic Science and Technology of China. This study was reviewed and approved by the Ethics Committee of Sichuan Provincial People’s Hospital (No.601). A clinical trial number was not applicable in this case. Since no care interventions were involved and all protected health information was removed before the analysis, written informed consent was waived by the Ethics Committee. We enrolled all OHCA cases that presented to the emergency department of our hospital between September 2023 and August 2024, as confirmed by the absence of a pulse, unresponsiveness, and apnea. We excluded patients under 18 years, those over 65 years, those with unwitnessed CA, those with incomplete medical records, and those who did not achieve return of spontaneous circulation (ROSC). The strengthening the reporting of observational studies in epidemiology (STROBE) guidelines was strictly followed (Supplementary File 1).

### Study variables

The study variables were collected from the medical records that included patients’ demographic characteristics, medical history, smoking history, CA/CPR location, CA/CPR details, neurological status on ICU admission, the cerebral gray-to-white matter signal intensity ratio (GWR), survival, the Cerebral Performance Category (CPC), and laboratory results (C-reactive protein (CRP), white blood cell (WBC) count, and monocyte (MONO) count). The primary outcome was the GWR after CA/CPR. The secondary outcomes were survival, the Cerebral Performance Category (CPC), and laboratory results.

### Quantification of the GWR

Previous studies have suggested that a loss of distinction between the gray matter (GM) and the white matter (WM), as assessed by computed tomography (CT), predicts poor neurological outcomes after cardiac arrest. The gray-to-white matter signal intensity ratio (GWR) was analyzed using brain CT images by a blinded investigator, following published methods (12, 13). Briefly, the CT scan was performed within 8 h after ROSC. A 10-mm^2^ circular area with a thickness of 5 mm was configured at the basal ganglia level, centrum semiovale level, and high convexity level. The GWR value was calculated as the average of both hemisphere values.

### Evaluation of the brain function

Based on previously published studies, we used the Cerebral Performance Category (CPC) to estimate the neurological outcomes. CPC 1 and CPC 2 were considered good cerebral performance and moderate cerebral disability, respectively. CPC 3–5 were classified as severe neurological disability, vegetative state, and death, respectively (14, 15).

### Statistical analysis

All data were analyzed using SPSS Version 25.0 (SPSS Inc., Chicago, IL, USA) by two authors independently, with discrepancies resolved by a third author. Quantitative variables were expressed as means and standard deviations, while qualitative data were expressed as numbers and percentages. Continuous variables were compared using two-tailed Student’s *t*-test. A chi-squared test was performed to compare the qualitative variables between two groups. Normal distribution of all continuous variables was tested using the Kolmogorov–Smirnov test. A *p*-value of less than 0.05 was considered statistically significant.

## Results

### Patient characteristics

We included 138 patients in our study. Of the included patients, 68 were subjected to CA/CPR during daytime (8:00 to 20:00) and the remaining 70 were subjected to CA/CPR during nighttime (20:00 to 8:00). There were no significant differences in any of the baseline demographics of patients subjected to CA/CPR during daytime and nighttime hours (Table 1), except for the CA/CPR locations. A higher proportion of patients subjected to CA/CPR was witnessed in public during daytime (69% vs. 34%), and a higher proportion of patients was at home during nighttime (66% vs. 31%, *p* = 0.039).

**Table 1:** Baseline characteristics of the study population according to the time of CA/CPR onset.

	Day (*n* = 68)	Night (*n* = 70)	*p*-value
Demographic characteristics			
Age, mean ± SD, years	50 ± 12	51 ± 14	0.861
Sex, male (%)	47(69)	51(73)	0.379
Weight, mean ± SD, kg	75 ± 11	71 ± 14	0.872
Medical history			
Hypertension, *n* (%)	38(56)	44(63)	0.266
Chronic heart diseases, *n* (%)	4(6)	6(9)	0.574
Diabetes, *n* (%)	6(9)	5(7)	0.733
Chronic respiratory diseases, *n* (%)	3(4)	6(9)	0.648
Smokers, *n* (%)	20(29)	26(37)	0.159
CA/CPR location			0.039
Home, *n* (%)	23(34)	48(69)	
Public, *n* (%)	45(66)	19(31)	
CA/CPR details			
Bystander-initiated CPR, *n* (%)	60(88)	59(84)	0.729
Time to basic life support, mean ± SD, minutes	6 ± 3	7 ± 3	0.873
Time to advanced life support, mean ± SD, minutes	9 ± 2	8 ± 4	0.794
Time to ROSC, mean ± SD, minutes	18 ± 7	20 ± 10	0.558
Intubation, *n* (%)	28(41)	27(39)	0.694
Neurological status on ICU admission			
GCS, mean ± SD	4 ± 2	4 ± 3	0.882
DOSE OF EPINEPHRINE, mean ± SD, μg/kg/min	0.07 ± 0.1	0.08 ± 0.1	0.749

### Evaluation of brain injury after CA/CPR

**Figure 1:**
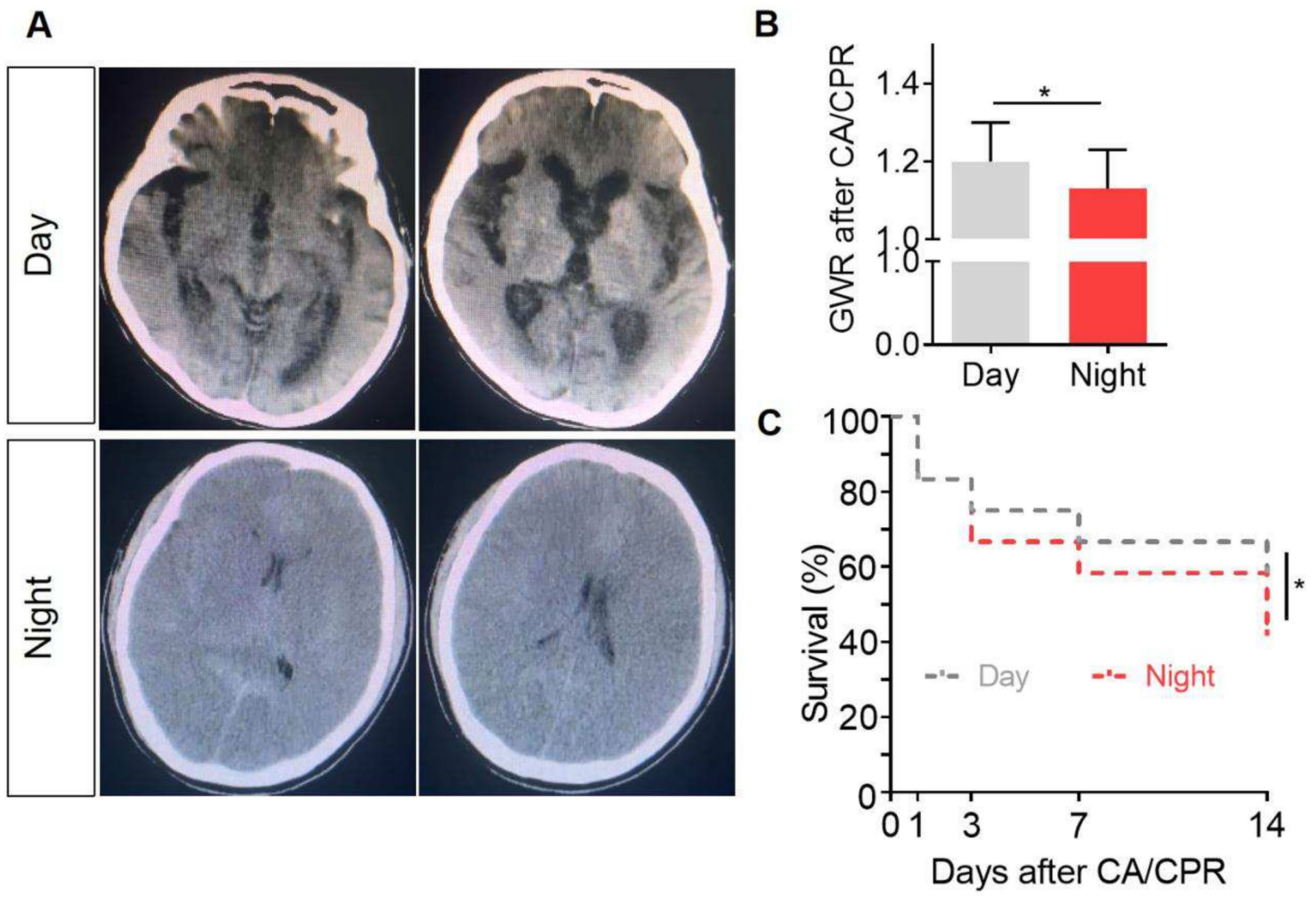
Evaluation of brain injury after CA/CPR. **(A)** Representative brain computed tomography (CT) images of the patients after CA/CPR. The brain CT scans that showed signs of more severe brain injury revealed a greater loss of gray–white matter differentiation, which was reflected by a smaller GWR value. **(B)** Gray–white matter ratio (GWR) values were quantitatively analyzed (1.20 ± 0.07 vs.1.13 ± 0.07, *p* = 0.035, effect size = 0.24). **(C)** Survival rate of the patients with different CA/CPR onset times. *p < 0.05.

The CT scan results indicated that GWR values were significantly lower in patients subjected to CA/CPR during nighttime compared to those treated during daytime (1.133 vs. 1.203, *p* = 0.036) (Figures 1A,B). Consistently, lower survival rates were also observed in nighttime CA/CPR survivors (Figure 1C).

In addition, the CPC results demonstrated that a greater number of patients subjected to CA/CPR during daytime were rated as class 1–2 on day 3, day 5, and day 7 after ROSC. In contrast, a larger proportion of CA/CPR survivors in the nighttime group were rated as class 5 on day 3, day 5, and day 7 after ROSC. Additionally, patients from both the groups received class 3–4 ratings as well (Table 2). Since lower GWR and higher CPC scores predict more severe brain injury (12), these results indicate that CA/CPR occurring at nighttime may contribute to poorer neurological outcomes.

**Table 2:** Cerebral performance after CA/CPR.

	Day (*n* = 68)	Night (*n* = 70)	*p*-value
Day 3			
1–2, (%)	20(29)	11(16)	0.036
3–4, (%)	31(46)	35(50)	0.874
5, (%)	17(25)	24(34)	0.043
Day 5			
1–2, (%)	25(37)	15(21)	0.012
3–4, (%)	23(34)	27(39)	0.176
5, (%)	20(29)	28(40)	0.021
Day 7			
1–2, (%)	29(43)	20(29)	0.019
3–4, (%)	17(25)	20(29)	0.282
5, (%)	22(9)	30(42)	0.027

### Comparison of blood-based biomarkers for brain injury after CA/CPR

Since certain blood-based biomarkers, including C-reactive protein (CRP), white blood cell count (WBC), and monocyte count (MONO), have been shown to be negatively associated with neurological outcomes after brain injury (16–18), we compared the results of these laboratory tests between the two groups. Elevated levels of CRP (11.3 ± 4.4 vs. 15.2 ± 6.7, *p* = 0.031), WBC (12.1 ± 3.9 vs. 15.9 ± 5.5, *p* = 0.025), and MONO (0.97 ± 0.19 vs. 1.21 ± 0.22, *p* = 0.043) were observed among nighttime CA/CPR survivors (Table 3). These results suggest that elevated levels of CRP, WBC, and MONO in the plasma after CA/CPR may contribute to poorer neurological outcomes.

**Table 3:** Comparison of the blood-based biomarkers for brain injury after CA/CPR.

Biomarkers	Day (*n* = 68)	Night (*n* = 70)	*p*-value
CRP (mg/L)	11.3 ± 4.4	15.2 ± 6.7	0.031
WBC (×10^−9^/l)	12.1 ± 3.9	15.9 ± 5.5	0.025
MONO (×10^−9^/l)	0.97 ± 0.19	1.21 ± 0.22	0.043

## Discussion

In this study, poorer neurological outcomes were observed in patients subjected to CA/CPR during nighttime (20:00–8:00) compared to those subjected to CA/CPR during daytime (8:00–20:00). These findings suggest new research directions that may help yield optimal outcomes in improving brain injury after CA/CPR.

Circadian rhythms are ubiquitous and have been implicated in a wide variety of clinical conditions (19). Previous studies have demonstrated that circadian rhythms affect infarction volumes after ischemic stroke in both patients and mice (9, 10, 20). Brain I/R injury is caused by the initial ischemia and subsequent reperfusion of the brain. Similarly, both ischemic stroke and CA/CPR can induce brain I/R injury after resuscitation. Therefore, we sought to determine whether circadian rhythms affect neurological outcomes after CA/CPR. Previous studies have confirmed the impact of circadian rhythms on in-hospital cardiac arrest (IHCA); however, whether circadian rhythms affect neurological outcomes after OHCA remains unexplored (21). Consistently, both the imaging data analysis and the CPC results revealed more severe brain injury in the patients with CA/CPR onset at nighttime compared with those with the onset at daytime. The results help expand our knowledge about the impact of circadian rhythms on brain I/R injury.

Previous studies have indicated that cardiac arrest often occurs unwitnessed at home and during the night (22–29). In this study, we found that the patients with CA/CPR onset during nighttime were more likely to experience worse neurological outcomes, which may be influenced by the CA/CPR location. However, this might be a biased and unreliable estimate. A published study identified the molecular mechanisms through which diurnal rhythms influenced neurological outcomes in a mouse model of ischemic stroke (9); however, the underlying mechanisms explaining the impact of CA/CPR onset time on neurological outcomes still need further exploration.

Several blood-based biomarkers, including CRP, WBC, and MONO, can help evaluate a patient’s prognosis following central nervous system injury. Higher levels of these biomarkers indicate increased inflammation (17, 18). In this study, we found that the plasma CRP, WBC, and MONO levels were higher among patients with CA/CPR onset at nighttime compared with those who experienced CA/CPR during daytime. This suggests that there is increased systemic inflammation after CA/CPR at night, accompanied by more severe brain injury.

This study has several strengths. First, the inclusion of patients with clearly defined CA/CPR onset time helped demonstrate the association between CA/CPR onset time and neurological outcomes. Second, the use of quantitative brain imaging data allowed us to more directly assess the diurnal effects on neurological outcomes after CA/CPR. There are also some limitations in this study that should be acknowledged. First, we only evaluated the survival rates and CPC score within 14 days and 7 days after CA/CPR. The long-term effect of circadian rhythms on CA/CPR-induced brain injury was not tested due to limitations in the medical records. Second, the study exclusively included OHCA patients; therefore, the results are only applicable to this group. Third, there could be other confounding factors that may affect the outcomes, such as the quality of CPR and the individual who performed it. However, we could not find this information in the medical records. Finally, although the time from collapse to identification likely varies between day and night, there was no significant difference between the two groups in terms of the time to basic life support, time to advanced life support, or time to ROSC.

## Conclusion

In conclusion, our study identified that the diurnal rhythm significantly influence neurological outcomes after CA/CPR. Patients with CA/CPR onset at daytime had better neurological outcomes compared to those with CA/CPR onset at nighttime. These findings highlight that the diurnal rhythm could serve as a potentially significant strategy for preventing and treating brain injury after CA/CPR.

## Data Availability

The raw data supporting the conclusions of this article will be made available by the authors, without undue reservation.
